# Excitation of nonradiating magnetic anapole states with azimuthally polarized vector beams

**DOI:** 10.3762/bjnano.9.139

**Published:** 2018-05-17

**Authors:** Aristeidis G Lamprianidis, Andrey E Miroshnichenko

**Affiliations:** 1 Nonlinear Physics Centre, The Australian National University, Canberra, ACT 2601, Australia; 2 Department of Mathematics and Applied Mathematics, University of Crete, 70013 Heraklion, Crete, Greece; 3 School of Engineering and Information Technology, University of New South Wales, Canberra, ACT 2600, Australia

**Keywords:** anapole excitation, dielectric nano-optics, multipolar expansion, T-matrix method, vector beams

## Abstract

Nonradiating current configurations have been drawing the attention of the physics community for many years. It has been demonstrated recently that dielectric nanoparticles provide a unique platform to host such nonradiating modes, called “anapoles”. Here we study theoretically the excitation of such exotic anapole modes in silicon nanoparticles using structured light. Alternative illumination configurations, properly designed, are able to unlock hidden behavior of scatterers. Particularly, azimuthally polarized focused beams enable us to excite ideal anapole modes of magnetic type in dielectric nanoparticles. Firstly, we perform the decomposition of this type of excitation into its multipolar content and then we employ the T-matrix method to calculate the far-field scattering properties of nanoparticles illuminated by such beams. We propose several configuration schemes where magnetic anapole modes of simple or hybrid nature can be detected in silicon nanospheres, nanodisks and nanopillars.

## Introduction

The triumph of Maxwell’s equations is associated with the unification of electric and magnetic phenomena on the same footing, which has led to the prediction of existence of an electromagnetic radiation, which can carry energy and momentum. As it is explained in all classical textbooks [1], any accelerated motion of a charged particle should result in the excitation of electromagnetic waves propagating away from the source, leading to radiative decay. Despite the success at the macroscopic scale, it fails when one tries to apply it at the microscopic level and attempts to explain the stability of an atom, where the electrons are confined and in constant motion, so they are inevitably accelerated. In quantum mechanics it was postulated that electrons live at certain orbits, which do not radiate. And this postulate was taken for granted, since it does not contradict any experimental observation at the microscale. But still, there were numerous attempts to find confined classical trajectories of charged particles that do not produce far-field radiation, and many interesting examples are known by now [2–6]. The key ingredient in their explanation is destructive interference in the far-field.

Despite the purely fundamental aspect of them, nonradiating sources have attracted a great deal of attention in various nanoscale structures. Recently, it was suggested that silicon nanodisks can support a nonradiating excitation known as anapole [7], literary meaning “without poles”, in which the electric dipole radiation vanishes completely. It was demonstrated that such states can be explained by the co-existence of electric and toroidal dipole moments, simultaneously excited inside dielectric nanoparticles. Despite their different near-field configurations both dipole moments emit with the same radiation pattern allowing for the complete destructive interference in the far-field. Toroidal dipole moments became extremely popular in the metamaterial community [8–9], which focused on designing optimal structures for their strong excitation. It turns out that the superposition of comparable toroidal and electric dipoles might result in nonradiating anapole excitation [10–14]. It has a number of interesting features and can be used to design near-field laser [15], to obtain high-efficiency harmonic generation [16], to achieve pure magnetic dipole scattering without admixture of other components under plane-wave illumination [17] as well as for a variety of other promising applications [18–19].

One may wonder now: Can nanostructures be a platform of hosting such nonradiating current configurations? The answer is yes. Actually, particular designs have been proposed and fabricated that experimentally verified the excitation of anapole states either in microwaves [20–21] or at optical frequencies [7,22]. One convenient way of approach is to analyze the scattered field into a superposition of vector spherical harmonics (VSHs). Due to their completeness and orthogonality any radiation profile can be uniquely decomposed in a series of such partial waves. It turns out, that high-index dielectric particles can exhibit infinite number of conditions where the scattering contribution of any such partial wave vanishes. As was mentioned above, the first zero of the electric dipole scattering, for example, can be explained in terms of destructive interference of Cartesian electric and toroidal dipoles. But for higher-order zeros additional terms are required and currently there is an effort to identify them all [23]. As highlighted in [24], according to the reciprocity theorem, a nonradiating anapole current configuration implies a poloidal current distribution that is orthogonal, in an integral manner, to the incident E-field distribution inside the volume of the particle. Moreover, it has also been proved that a nonradiating current configuration will bear no spatial frequencies that are able to be coupled with free space radiation [25]. In that sense, a multipolar anapole condition corresponds to a current configuration that has a spatial frequency spectrum on the spherical cell of radius k_0_, that is orthogonal to the far field radiation of that particular multipolar partial wave. Recently, the excitation of anapole states in pure dielectric nanoparticles was studied under the prism of a Weierstrass-type product expansion in terms of the resonant-state frequencies that correspond to zeros of the elements of the S-matrix of the particle in the complex frequency plane [26]. Moreover, excitation of anapole states has also been examined under a projection scheme on Fano–Feshbach resonances [27]. For our purposes, we associate any zeros of the partial scattered power of spherical harmonics with the potential of nonradiating anapole excitation.

Importantly, high-index dielectric nanoparticles, in addition to multipolar modes of electric type, also support magnetic ones. In analogy with anapole excitations of electric type, one might also expect the existence of magnetic anapoles, associated with the vanishing scattering contribution of the magnetic dipole term. It now raises the question of how such conditions can be experimentally observed. The problem is that under plane-wave illumination such vanishing partial wave scattering will usually be overshadowed by stronger contributions of other modes. The answer to this question was recently suggested by a number of groups and incorporates the use of so-called structured-light excitations. Lately, vector beams generally attracted lots of scientific attention since they can have various interesting applications [28–31]. With such illumination schemes we are able to shape the multipolar content of the incident field. For example, by using a radially polarized focused beam, only spherical harmonics of electric type will be present at the focal point of the beam, without any magnetic. It was suggested that such a type of illumination offers an ideal condition for the electric anapole excitation, which can be tested experimentally [24,32]. It turns out, that if we change the polarization from radial to azimuthal, then, only magnetic harmonics will be present. Thus, using such an illumination scheme will be ideal to test the possibility of the magnetic anapole excitation. An excited magnetic anapole is expected to have useful applications in biosensing, i.e., in the detection of molecules that interact strongly once exposed to magnetic field hotspots, which nanoparticles in a magnetic anapole state can offer in their near field. Moreover, the signal-to-noise ratio of an MRI machine, that is defined as the ratio of the local magnetic to electric field intensity, could be significantly improved by employing these nano-optical properties that a magnetic anapole can offer.

In this paper we discuss in detail the possibility of magnetic anapole excitation under various configurations and suggest how they can be tested experimentally. We begin with a general multipolar analysis in vector spherical harmonics of arbitrary focused beams. We generalize the results of [33–34] for arbitrary focused beams under 2π- or 4π-illumination schemes and for arbitrarily translated frames of reference compared to the coordinate system of the beam. Next, we describe the T-matrix method that we use to treat the scattering process of nanoparticles under arbitrary illumination schemes and we highlight important properties of the T-matrix under several symmetry conditions. We also discuss the implications that an anapole excitation condition would have on the coupling of the external field with the natural modes of the nanoparticle under the prism of the singular value decomposition of its T-matrix. Last, we present our results on the excitation of magnetic anapole states under various illumination schemes and various geometries of hosting nanoparticles.

## Multipolar Decomposition of Focused Beams

Physicists have been lately concerned with the multipolar decomposition of various types of structured light, in order to study its interaction with particles [33–37]. Here, we consider a focused beam propagating along 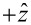, with *x*O*y* being its focal plane. An arbitrary paraxial beam, with sufficiently large waist in order to neglect its longitudial *z*-component, can be considered as a superposition of one radially (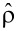) and one azimuthally (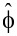) polarized beam with different complex transverse profiles for each one of this parts. According to [38], such a beam, after being focused by a lens, has an electric field intensity that is given near its focal point, with respect to the O(**r**) coordinate system, by the following formula:

[1]
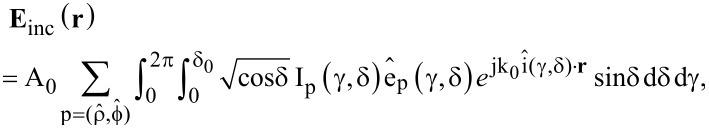


where 
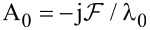
 is a constant, with 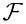 being the focal length of the objective and λ_0_ the free-space wavelength. k_0_ is the correspondent wavevector. δ_0_ is the angle of the marginal ray that passes through the entrance pupil of the objective, which has a numerical aperture of NA. So, δ_0_ = sin^−1^(NA). The term 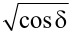 derives from the conservation of the energy flux of each ray that is refracted through the Gaussian reference sphere of the objective. The indicator p stands either for the radial (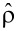) or the azimuthal (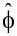) part of the input beam. The field at the focal plane of the objective is a kind of 2D inverse Fourier transform of the input to the lens field. So, in that sense, it is expressed as a superposition of plane waves propagating in angles γ,δ, with γ being the azimuthal and δ the polar angle of the direction of the propagation vector of each plane wave. Each pixel of the input to the objective field with cylindrical coordinates (
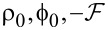
) gives birth to a plane wave that propagates along the direction


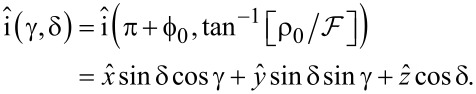


The radial part of the E-field of such a pixel gives birth to a plane wave in the image space that is polarized along


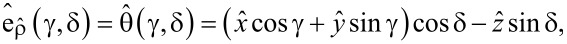


whereas the azimuthal part of the E-field of such a pixel gives birth to a plane wave polarized along


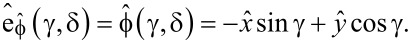



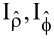
 are the correspondent complex transverse profiles of the input beam that impinges on the objective.

Next, we decompose the E-field of such a focused beam into an expansion of vector spherical harmonics with respect to a coordinate system O_1_(**r**_1_), the natural frame of the scatterer, that is translated and parallel to the coordinate system O(**r**) of the focused beam. The position vector of its center O_1_, in spherical coordinates, with respect to the coordinate system of the focused beam, is given by the vector **d**(R,Θ,Φ) = **r**− **r**_1_.

[2]
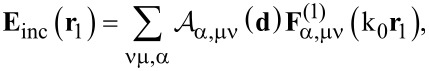


where 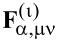 are the VSHs. The indicator α acquires the names M,N for the TE (magnetic) and TM (electric) VSHs respectively, the index ν stands for the angular momentum quantum number, which takes the values 1, 2, … and corresponds to dipoles, quadrupoles, etc. The index μ stands for the azimuthal quantum number, which takes the values −ν, …, −2, −1, 0, 1, 2, …, ν. The exponent (ι) refers to the correspondent Bessel (ι = 1, 2) and Hankel (ι = 3, 4) functions of first and second kind, respectively. For the incident field, being a standing wave, we make use of the Bessel functions. The VSHs are given by the formulas below [39]:

[3]
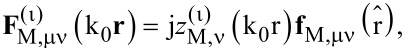


[4]
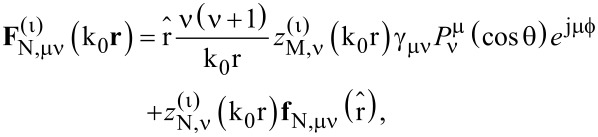


where

[5]
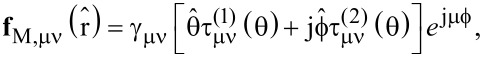


[6]
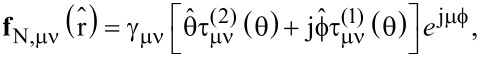


and 
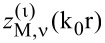
 is a spherical Bessel (ι = 1) or Hankel (ι = 3) function of the first kind and


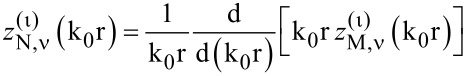


is the corresponding Riccati function. 
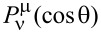
 is the associated Legendre function of the 1st kind, with


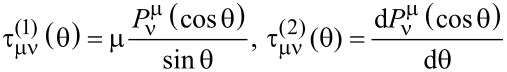


being the generalized Legendre functions.


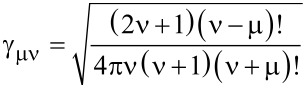


is a normalization factor for the Legendre functions and plays an important role for the numerical calculation of the elements of the T-matrix that will follow later.


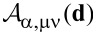
 are the correspondent spherical amplitudes of the multipolar expansion of the incident field with respect to the coordinate system O_1_(**r**_1_). In order to calculate them we make use of the above plane wave spectrum representation of such an excitation field. In our case they are straightforwardly given by the formula:

[7]
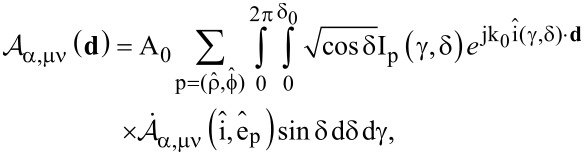


where we substituted

[8]
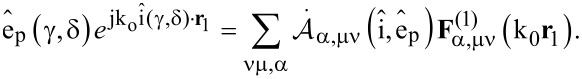


It is proven (see Supporting Information File 1) that the spherical amplitudes of the multipolar expansion of each component of the plane wave spectrum of the incident field, denoted as 
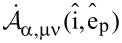
, are given by the formula below:

[9]
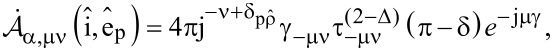


where 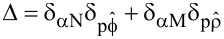 and δ_αβ_ is the Kronecker delta, which yields 1 for α = β, or 0 for α ≠ β.

By integrating the flux of the Poynting vector of the incident field over an infinite plane, oriented transverse to the optical axis, we can calculate the total power that such a focused beam carries. It is given by the formula below:

[10]
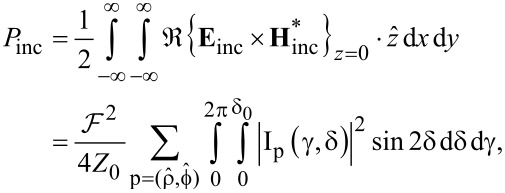


where *Z*_0_ is the wave impedance of free space.

At this point, we would also like to consider the case of two counterpropagating focused beams that constitute a standing wave excitation. For simplicity, without loss of generality, we consider that both beams are focused by the same optical system. The newly introduced beam travels towards the 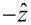 direction and is described by the coordinate system 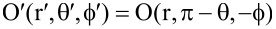. By applying the same rotation transformation to the natural frame of the scatterer and making use of properties of the Legendre functions, we have


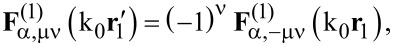


where, again, the rotated system attached to the natural frame of the scatterer is denoted with a prime. We end up with the following expression for the spherical amplitudes 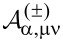 that correspond to the multipolar decomposition of the superimposed counterpropagating beams around the natural frame of the scatterer O_1_(**r**_1_):

[11]
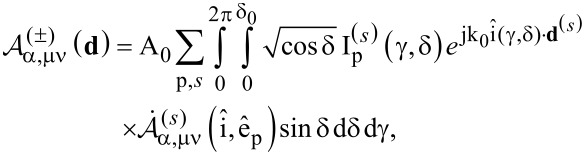


where

[12]
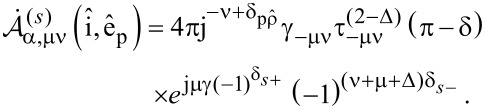


The indicator *s* stands for the propagating direction of the two beams and takes the values + and − for the beam that propagates along the 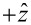 and 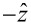 direction, respectively. The complex transverse profile of the first beam with respect to the O(**r**) coordinate system is denoted as


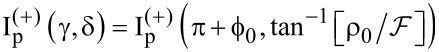


and the complex transverse profile of the second beam with respect to the O′(**r**′) coordinate system is denoted as


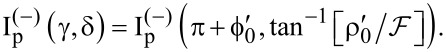


Moreover, the spherical coordinates of the position vector with respect to the O(**r**) coordinate system for the two cases are **d**^(+)^ = (R, Θ, Φ) and **d**^(−)^ = (R, π − Θ, −Φ), respectively.

To access some of the properties of the above multipole expansion of the excitation field, we further simplify the formula by expanding the complex transverse profiles of the beams into Fourier series with respect to the azimuthal angle γ. So, due to its 2π-periodicity, we have: 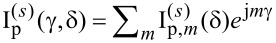, which is an expansion into modes with different orbital angular momentum *m*. Then, we can also perform the integration over the azimuthal angle analytically. This yields the simplified formula of Equation 13 where *J*_ν_(*x*) is the cylindrical Bessel function of first kind.

[13]
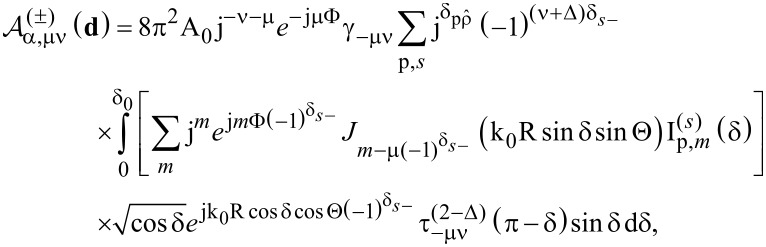


We can observe that in the case of sinΘ = 0, which means that the natural frame of the scatterer is located along the optical axis of the two beams, the argument of the Bessel function becomes zero, and therefore we end up with the condition 
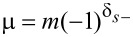
. This implies that the orbital angular momentum *m* of the input beams before their focusing is bequeathed to the azimuthal quantum number of the multipolar expansion of the focused beams with respect to such a reference frame. This would mean, for example, that a focused beam with *m* = 3 would bear only multipoles of orders higher than ν = 2. It would lack a dipolar and quadrupolar content.

We conclude this section by considering the particular case of rotationally symmetric (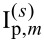 = 0 for *m* ≠ 0) focused beams with the natural frame of the scatterer being along the optical axis (sinΘ = 0). The spherical amplitudes of the field that corresponds to such a case are given by Equation 14.

[14]
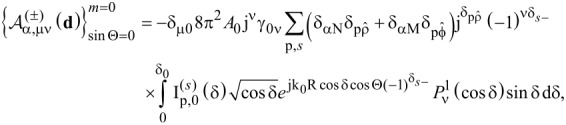


In Equation 14 we want to place emphasis on the following observations:

(1) The radial part of the beams bears a multipolar content of purely electric type, whereas the azimuthal part bears a multipolar content of purely magnetic type.

(2) Due to the mirror symmetry with respect to the focal plane, if the two counterpropagating beams have the same complex transverse profile, 
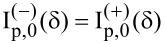
, then, for a natural frame of the scatterer placed at their common focal point (R = 0), the excitation field has a multipolar content of purely even order ν. In contrast, if the two counterpropagating beams are out of phase, 
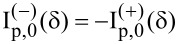
, then the excitation field has a multipolar content of purely odd order ν, for a natural frame of the scatterer placed at the focal point (R = 0).

To sum up, in this section we began with the plane-wave representation of a focused beam, which is the decomposition of its field into the Cartesian eigenfunctions of the Helmholtz equation of free space. Based on this, we derived formulas for its multipolar decomposition in VSHs, which is the decomposition of its field into the spherical eigenfunctions of the Helmholtz equation of free space. This second expansion, as we will see in the next section, is of crucial importance in studying the far-field scattering properties of particles illuminated by such an excitation field.

## T-Matrix Method for Far-Field Scattering Calculations

The T-matrix formulation is a general method of dealing with far-field scattering phenomena. It is based on decomposing both the incident and the scattered field into the basis of the eigenfunctions of the Helmholtz equation of free space. So, both fields can be represented by vectors containing their correspondent expansion amplitudes. Thinking in terms of a multichannel system where the input is the incident field and the output is the scattered field, the scattering process is a linear process that can be described by a square matrix, called the T-matrix, which takes as an input the amplitudes of the incident field and gives as an output the amplitudes of the scattered field. The decomposition of the fields could be performed either on a Cartesian eigenfunction basis, that is in plane waves, or in a spherical eigenfunction basis, that is in VSHs. The plane-wave spectrum is a continuous spectrum that needs to be discretized, whereas the VSHs spectrum is a discrete but infinite spectrum. The great advantage of using VSHs as the basis of those decompositions is that the rows and columns with significant elements of the T-matrix are boiled down to the first fundamental multipolar terms. This means that the infinite T-matrix can be truncated early without loss of accuracy. We end up fully describing the phenomenon of the EM-scattering just by a *N* × *N* matrix, where *N* = 2ν_max_(ν_max_ + 2), with ν_max_ being the maximum order of the multipolar decomposition of the fields that we will take under consideration. For subwavelength particles, ν_max_ can take values, for example, from 2 or 3 up to 15 or 20. This depends on (1) the size of the particle: small particles can be sufficiently described by just using the very first orders; (2) the geometrical complexity of the particle, that is, to which extent the particle is aspherical; and also (3) the degree of accuracy that we want to achieve. The absolute values of the elements of the T-matrix range from 0 to 1 and usually they decrease while the order of the multipoles they refer to increases. So, practically, the particle can only interact with incoming multipoles of the order of up to ν_max_. For higher-order multipoles it behaves as if it was transparent and this fact enables us to truncate the matrix.

One other significant feature of the T-matrix is that it is characteristic of the particle in the particular wavelength and it is irrelevant to the incident field. Once computed it can be straightforwardly used for scattering calculations from arbitrary excitation. Hence, one can understand that the T-matrix method plays a key role in capitalizing on the results of the previous section. The spherical amplitudes 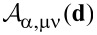 of the multipolar decomposition of the excitation field will compose the input vector of the multichannel system with a transfer function given by the T-matrix and the output being the vector of the spherical amplitudes of the multipolar decomposition of the scattered field, which will be denoted as 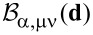. Shaping the light that is incident to the particle, that is, acting on the input vector of the system, instead of acting upon the inner dynamics of the system, i.e., its transfer function, the geometry of the particle, is an alternative way of designing the output of the system, i.e., the scattering response of the particle. The T-matrix method reveals the inner dynamics of the scattering system. By determining the multipolar content of the field scattered by the particle one has immediate knowledge of its far-field radiation pattern as well. This means that one readily knows its behavior as a nanoantenna. Hence, apart from being useful for analytical purposes, it is also a powerful synthetical tool when it comes to designing the scattering response of a particle. And this is a very special and valuable feature, that other methods of approaching scattering problems rarely offer.

The multipolar expansion of the scattered field with respect to the natural frame of the scatterer is given by:

[15]
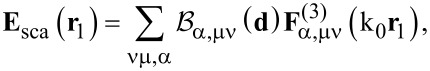


where we make use of the Hankel instead of the Bessel functions for the VSHs since the scattered field is a radiating field. The series of this formula is considered to converge only outside of the circumscription of the particle sphere, and this is known as the Rayleigh hypothesis [40–41]. The spherical amplitudes of the scattered field will be given in the T-matrix formulation by the following expression:

[16]
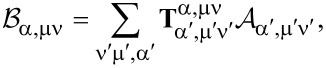


or in matrix formulation: **B** = **T**· **A**.

Not only the spherical amplitudes of the input vector, but also the elements of the T-matrix, as well as the values of the output vector of the scattered spherical amplitudes, do depend on the choice of the reference frame of the scatterer, i.e., the coordinate system that we will employ for the multipolar decomposition of the fields. There are analytical formulas to obtain a new version of the T-matrix of a particle that corresponds to a rotated or translated system of coordinates [39,42].

The total power that is scattered by the particle is given by integrating the flux of the Poynting vector of the scattered field over a spherical surface of infinite radius and is given by the formula:

[17]
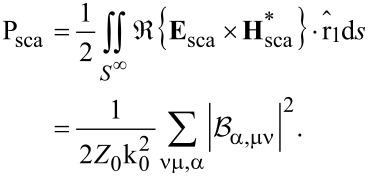


There are several methods in order to calculate the elements 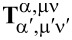 of the T-matrix [43–45]. We are going to employ the semi-analytical extended boundary condition method (EBCM) [39,46], also known as null-field method [42], which was originally introduced by Waterman in 1965 [47]. This method best applies for cases of homogeneous, isotropic, star-shaped particles. The elements of the matrix end up being given by Stratton–Chu type integrals, with the Dyadic Green functions being expanded in dyadic products of VSHs, over the surface of the particle that match the boundary conditions of the multipolar field representations inside and outside of the particle. This method is highly efficient for the cases of rotationally symmetric particles, such as cylinders, since the integration over the azimuthal angle can be performed analytically leading to simplified expressions of single integrals over the polar angle (see Supporting Information File 1). One needs to pay special attention on the fact that the EBCM method becomes numerically unstable for particles with extreme aspect ratios, due to numerical calculations with limited digits of precision [48–49]. This is mainly a problem because in these integrals Hankel functions with small arguments are involved, which give outputs that go to infinity and spoil the integration. Limited precision accuracy plagues also the inversion process of a matrix (see Supporting Information File 1) that is usually close to being singular and is needed for the calculation of the T-matrix.

The T-matrix has some important symmetry properties that need to be taken under consideration [39,42]:

(1) A particle that is rotationally symmetric with respect to the *z*_1_-axis of its natural frame has a T-matrix that is diagonal over the index μ, which means that the azimuthal quantum number of the scattered field is inherited by that of the incident field:


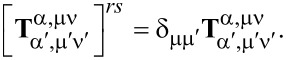


(2) A particle with *N*-fold symmetry with respect to the *z*-axis of the natural frame of the scatterer has the following property:


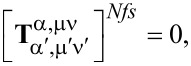


for |μ − μ′| ≠ κ*N*, with κ = 0, 1, … This means that a particle of small size can practically exhibit diagonality over the azimuthal index, which means behave like a rotationally symmetric particle, if it has a *N*-fold symmetry with *N* being greater than twice the maximum index ν_max_ where we truncate the T-matrix. For example, for a particle small enough, so that it practically interacts only with dipole fields (dipole approximation, ν_max_ = 1), a three-fold symmetry is enough for it to behave as a rotationally symmetric particle in terms of its far-field scattering [50].

(3) A particle that is rotationally symmetric with respect to the *z*_1_-axis of its natural frame has a T-matrix with also the following property:


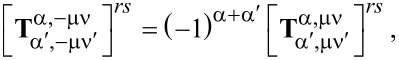


which gives


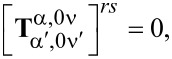


for α ≠ α′. This means that for a rotationally symmetric excitation, with μ′ = 0, the electric multipoles of the incident field give birth only to scattered multipoles of electric type, and the magnetic multipoles give birth only to magnetic multipoles.

(4) A particle with mirror symmetry with respect to the *z*_1_ = 0 plane of its natural frame has a T-matrix with the following property:


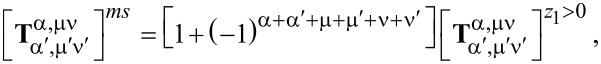


where with the last notation, integration over only half of the surface of the particle with *z*_1_ *>* 0 is implied. If the particle is rotationally symmetric as well, we have that


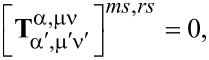


when α = α′ and ν + ν′ is an odd number, or when α ≠ α′ and ν + ν′ is an even number. This means that for a rotationally symmetric excitation (μ′ = 0), the incident multipoles of even order give birth only to scattered multipoles of even order, and, similarly, the incident ones of odd order, only to scattered ones of odd order.

(5) A particle with spherical symmetry has a T-matrix that is diagonal over all its indices α, μ, ν:


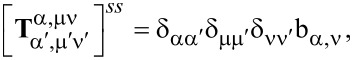


where b_α,ν_ are the well-known Mie scattering coefficients, which in the case of homogeneous spheres are given by the formula:

[18]
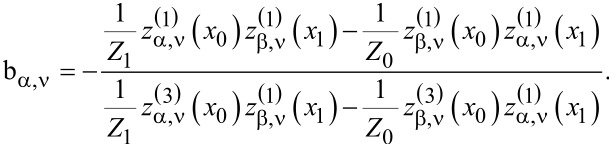


The indices α, β acquire the names M and N with α ≠ β. The abbreviations *x*_0_ = k_0_α_0_ and *x*_1_ = *n*_1_k_0_α_0_ are used, with α_0_ being the sphere radius and *n*_1_ its refractive index. *Z*_1_ is the wave impedance inside the particle and *x*_0_ is called the size parameter of the sphere.

We will close this section by unveiling the property that the T-matrix of one particle should have in order to be able to host a nonradiating anapole state. For this, we need to perform a singular value decomposition (SVD) of the T-matrix [51]:

[19]
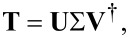


where Σ is a diagonal matrix with real, positive elements σ*_i_* sorted in descending order, **U** and **V** are unitary matrices with columns of the vectors **u***_i_* and **v***_i_*, respectively, and both form a different orthonormal basis in *N*-space. Those vectors are called the left- and right-singular vectors of the T-matrix and form pairs of vectors that correspond to the singular values σ*_i_* of the matrix. They have the property **T**· **v***_i_* = σ*_i_***u***_i_*.

An ideal anapole state, corresponding to zero scattered power, can only be hosted by a particle whose T-matrix has at least one singular value equal to zero. If this condition is satisfied, then the ideal anapole can be excited by illuminating it by a field that has a spherical wave amplitude vector **A**_0_ that can be written as a linear combination of the null-space vectors of the T-matrix, that is as a linear combination of the right-singular vectors that correspond to singular values equal to zero: 
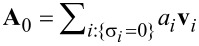
. Then we would have **B** = **T**· **A**_0_ = 
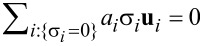
. So, this is the ideal condition of the T-matrix that would indicate the ability of the particle to host an ideal non-radiating anapole state. In order to have access to such kind of excitation we need to shape the multipolar content of the illumination field. The result is that the particle is critically coupled with the excitation field inside of which electromagnetic energy is stored without a scattered field needed to fulfill the boundary conditions on its surface. Furthermore, we should also denote that those right-singular vectors of the null-field space should describe nontrivial excitations, that is excitations with significant multipolar content for multipoles of the first order, since, for higher-order multipoles, a nanoparticle is actually behaving as if it was transparent. The incident field in the vicinity of the center of the coordinate system attached to the particle is mainly described by the first few multipolar terms.

However, in practice, when exciting an anapole state, we just aim to minimize, not to eliminate, the norm of the vector **B**. If we define the quality factor of the anapole excitation as *Q* = (4*P*_inc_/*P*_sca_) − 1, then, by expanding the output vector again on the basis of the left-singular vectors, finally we have:

[20]
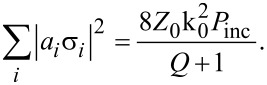


It becomes obvious now that the ability of a particle to host a nonradiating anapole state with high *Q*, in terms of its T-matrix, means the correspondence of sufficiently small coupling coefficients α*_i_* to the first singular values σ*_i_*, which take the largest values, since they are sorted in descending order. So, in order to achieve a nonradiating state, the key lies in illuminating the particle with a field, the multipolar decomposition of which, will be well described by a linear combination of those right-singular vectors that correspond to small enough singular values. We need to avoid using, for our input, the right-singular basis vectors that are not enough scaled down in magnitude by the T-matrix transformation.

## Results and Discussion

We can use the results and conclusions of the previous sections in order to design the scattering response of a nanoparticle at will. We want to achieve a purely magnetic response by the particle, the scattered power of which, will be suppressed for some particular frequency. We will properly design its geometry and, also, its excitation field, in order to achieve the desired scattering response.

In the former sections, we proved that an azimuthally polarized focused beam that is rotationally symmetric, i.e., that has a zero orbital angular momentum: *m* = 0, bears only multipoles of magnetic type for particles that are located over the optical axis. Moreover, it was also proven that if this particle has a rotational symmetry as well, it will only scatter multipoles of magnetic type. So, it will have a purely magnetic response. More specifically, the scattered field would be a superposition of VSHs of type 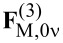, with the magnetic dipole, 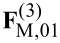, corresponding to a dipole moment along the *z*-axis, **m***_z_*, and the magnetic quadrupole, 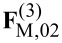, corresponding to a Cartesian quadrupole moment 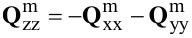, and so on. So, for demonstration purposes, we are going to use an azimuthally polarized, rotationally symmetric, focused vector beam with a Bessel–Gauss transverse profile given by


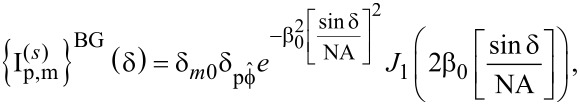


with a ratio of pupil radius to beam waist β_0_ = 1.5 and a numerical aperture of NA = 0.85. The multipolar decomposition of such an excitation in VSHs is given by Equation 14.

**Figure 1:**
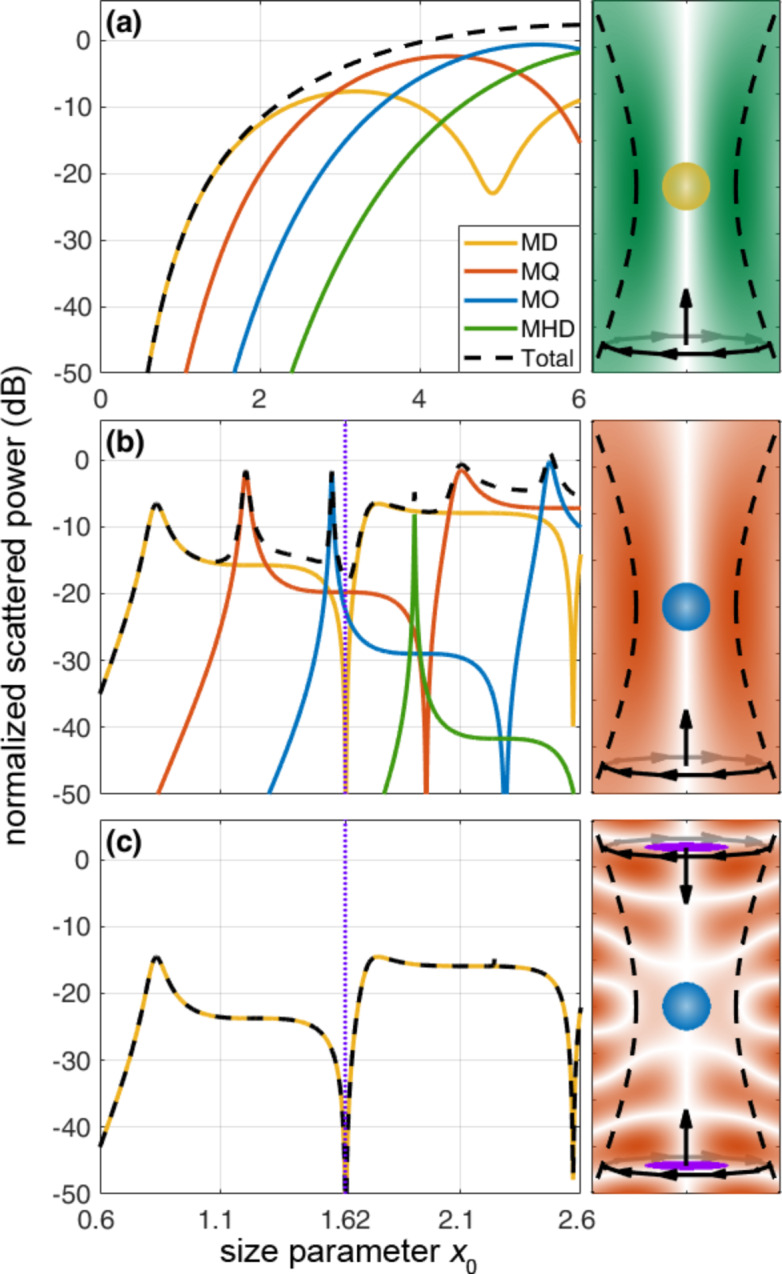
Plots of the normalized scattered power (*P*_sca_/*P*_inc_) in dB, decomposed into its multipolar contributions, as a function of the size parameter of the particle, *x*_0_. a) Golden sphere at the focal point of an azimuthally polarized, rotationally symmetric beam at λ_0_ = 500 nm. b) Silicon sphere at the focal point of an azimuthally polarized, rotationally symmetric beam at λ_0_ = 1550 nm. c) Silicon sphere at the focal point of two counterpropagating, out of phase, azimuthally polarized, rotationally symmetric beams, modulated by the proposed phase mask, at λ_0_ = 1550 nm. Purple dotted lines indicate the first anapole condition for a sphere of size *x*_0_ = 1.62. On the right-hand side, there are illustrations of the actual E-field intensity of the excitation over a window of 2 × 4 wavelengths at the focal region, on the ρO*z* plane, with a sphere of size *x*_0_ = 1.62 placed at the focal point.

In Figure 1a we plot the normalized total scattered power, (*P*_sca_/*P_inc_*), at a wavelength of λ_0_ = 500 nm, for golden spheres of various sizes, placed at the focal point of a vector beam like the one described above. As shown there, in such a way, we can even achieve a purely magnetic response by a plasmonic nanoparticle at optical frequencies. Of course, gold has a poor performance in optical frequencies, since it suffers from severe thermal losses, and as a result, the resonances of the particle correspond to Mie scattering coefficients, the amplitudes of which, are usually significantly less than one, and the quality factor of the supported anti-resonances is quite low as well. A plasmonic nanoparticle can only provide us with a very poor (anti-)resonant spectrum to work with.

So, we resort to high-index silicon nanoparticles as a platform to excite a nonradiating anapole state of magnetic type. Silicon nanoparticles suffer negligible losses at infrared light. Due to their high refractive index, they constitute also a good platform to host Mie resonances of magnetic type, since they can confine light and support circulating current loops inside. Now, we replace the golden sphere with a one of silicon and in Figure 1b, we plot the normalized total scattered power (*P*_sca_/*P*_inc_) at a wavelength of λ_0_ = 1550 nm, for spheres of various sizes. As we can see, its resonant multipolar content is much richer than the previous case of the plasmonic nanoparticle. We can observe a steep dip of the scattered power for a sphere of size parameter *x*_0_ = 1.62, which belongs to a sphere of a radius of α_0_ = 400 nm. We have already avoided any interference by the electric modes and this sphere could be a promising candidate to host a magnetic anapole state, if it were not, as we can see, for the overlapping magnetic quadrupole and octupole that interfere and spoil the anapole condition of the magnetic dipole.

To overcome this, we take the following two actions: First, we employ a second, similar, counterpropagating beam and illuminate the silicon particle under a standing-wave configuration. As proven in the previous section, if the second counterpropagating beam has the same transverse profile and is out of phase with the first beam, there would be no excitation of multipoles of even order for a scatterer located at their common focal point. And, since the T-matrix of a spherical particle is diagonal over ν, the scattered multipoles of even order are canceled out. Therefore, there will be no spurious interference by the magnetic quadrupole any more. But we still need to get rid of the octupolar interference. For this, we apply a phase mask to the two beams before their focusing by the objective. The simplest phase mask that we can come up with is introducing a π phase difference inside a circular disk with a radius that corresponds to the angle δ where the cumulative value of the integral of Equation 14 corresponding to the magnetic octupole term takes half of its final value. In our case, this happens for δ = 29.225°. In this way, the octupolar content of the beam is eliminated, finally leading to the excitation of a nearly ideal magnetic anapole state that has a dynamic range of more than three orders of magnitude (Figure 1c). Only spherical particles with perfect dielectric behavior or of perfect conductivity can exhibit truly ideal multipolar dips to zero. We should also note the fact that any silicon nanosphere placed at the focal point of such illumination scheme will exhibit purely magnetic dipole response. However, as one can observe, there is also a price to pay for the phase mask that we introduced: the field intensity at the vicinity of the focal spot drops to less than a third. Nonetheless, one could potentially come up with more efficient ways of shaping the multipolar content of the excitation, i.e., more complex illumination schemes that can also include amplitude modulation or multiple-beam configurations. Alternatively, avoiding the use of such a phase mask, we can observe that one can obtain another anapole corresponding to the magnetic quadrupole of a sphere of size *x*_0_ = 1.96. Under illumination of two in phase beams the interfering magnetic dipole and octupole will be diminished, leading to a scattering dip of less than −30 dB between two, hexadecapolar and quadrupolar, resonances. In Supporting Information File 1, one can find a plot with the proposed phase-modulation mask, together with some electric and magnetic field plots that correspond to the illumination of a silicon sphere, with the size of this anapole case, under illumination of both one single and two out of phase beams, with and without the phase mask applied.

**Figure 2:**
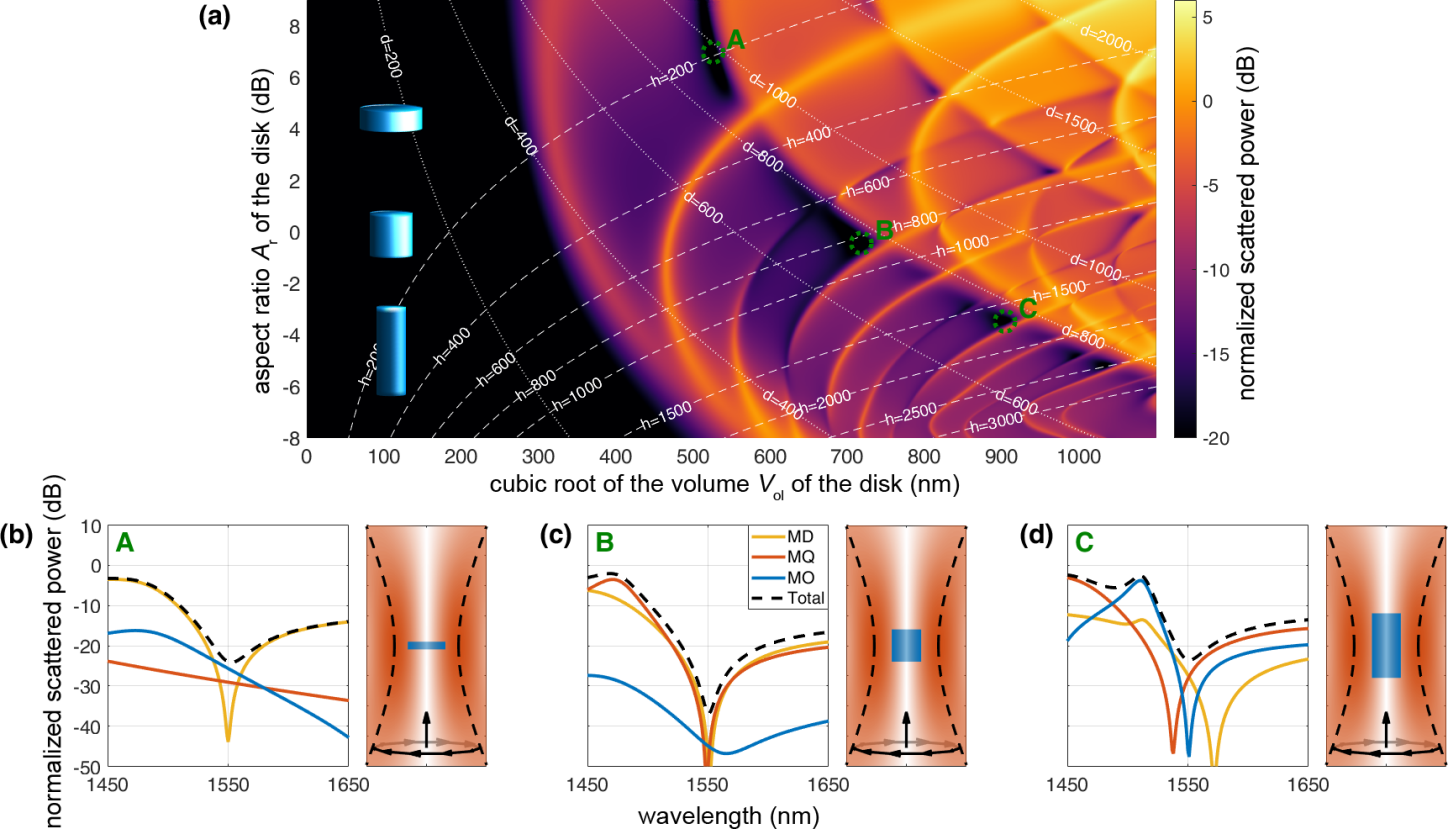
a) 2D plot of the normalized scattered power (*P*_sca_/*P*_inc_) in dB as a function of the cubic root of the volume *V*_ol_ of the disks and the aspect ratio *A*_r_ of the disks. Isoheight (dashed) and isowidth (dotted) contour lines in terms of the dimensions of the disks (in nm) are plotted as well. The silicon disks are located at the focal point of a rotationally symmetric, azimuthally polarized vector beam at λ_0_ = 1550nm. Three cases of excited anapole states are highlighted with A, B and C, the multipolar decomposition of the scattering spectrum of which, is depicted in subfigures b-d respectively. On the right side of them, there are illustrations of the actual E-field intensity of the excitation over a window of 2x4 wavelengths at the focal region, with a disk of the corresponding dimensions placed at the focal point.

Next, we will shift our attention to silicon disks since they represent, in terms of fabrication, experimental verification and applications, more realistic cases. We use the T-matrix theory described before in order to study numerically the interaction of an azimuthally polarized cylindrical vector beam with the cylinders. As one can see in Figure 2a, we scan the aspect ratio, *A*_r_ = *d*/*h*, and the volume 
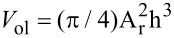
 of various cases disks , with diameter *d* and height *h*, located in free space illuminated by a single azimuthally polarized, cylindrically symmetric, focused vector beam at fixed wavelength λ_0_ = 1550 nm, plotting the normalized total scattered power (*P*_sca_/*P*_inc_) on a logarithmic scale in search of dips that would indicate the presence of some anapole states. The disks are aligned along the optical axis.

The particular design of the excitation is essential in obtaining nonradiating behavior of the cylinders. Again, we want to emphasize that the symmetries of the cylinder are of crucial importance. Such a particle is rotationally symmetric, which means that it will provide the diagonality identity over the index μ, which is zero due to the cylindrical symmetry of the excitation. This will result in the scattered field being of purely magnetic type. In addition, a cylinder exhibits a mirror symmetry with respect to the *z* = 0 plane. This property, as we have shown earlier, will cancel out all scattered multipoles of even order when using the same standing-wave configuration that was employed before for the silicon sphere. This is the case even if the T-matrix of a cylinder disk does not have this diagonality over the index ν that spherical particles have. However, as we can see in Figure 2a, there are several anapole instances already accessible by the single beam illumination scheme. In Figure 2b–d, we focus our attention on three of these cases. The first anapole case is hosted in an oblate disk of small volume, *V*_ol,A_^(1/3)^ = 526 nm, but with high aspect ratio, *A*_r,A_ = 4.98. It is a magnetic anapole that exhibits a single dip of the magnetic dipole. The two other cases, that appear in larger disks of bigger volumes, *V*_ol,B_^(1/3)^ = 718 nm and *V*_ol,C_^(1/3)^ = 904 nm, but with smaller aspect ratios, *A*_r,B_ = 0.905 and *A*_r,C_ = 0.45, respectively, exhibit magnetic anapole states of higher order. There is a hybrid condition where, simultaneously with the magnetic dipole dip, we also have dips of the magnetic quadrupole and of the magnetic octupole. In Supporting Information File 1, there are 2D maps of the multipolar decomposition that corresponds to Figure 2a.

Last, we compare the properties of the T-matrix of the cylinder for which the second anapole case was highlighted previously, with the properties of the T-matrix of a cylinder that has the same aspect ratio, *A*_r,B′_ = *A*_r,B_ = 0.905, but a larger volume, *V*_ol,B′_ = 759 nm, and yields resonant scattering. We perform a singular value decomposition of the μ = 0 T-submatrices for each of those two cylinders and calculate their singular values σ*_i_*. We also expand the input vector **A**, with the spherical amplitudes of the excitation field, over the basis of the right-singular vectors of each matrix, calculating the coupling coefficients *a_i_* for both of the two cases. We show the corresponding results for multipoles up to the fifth order.

**Figure 3:**
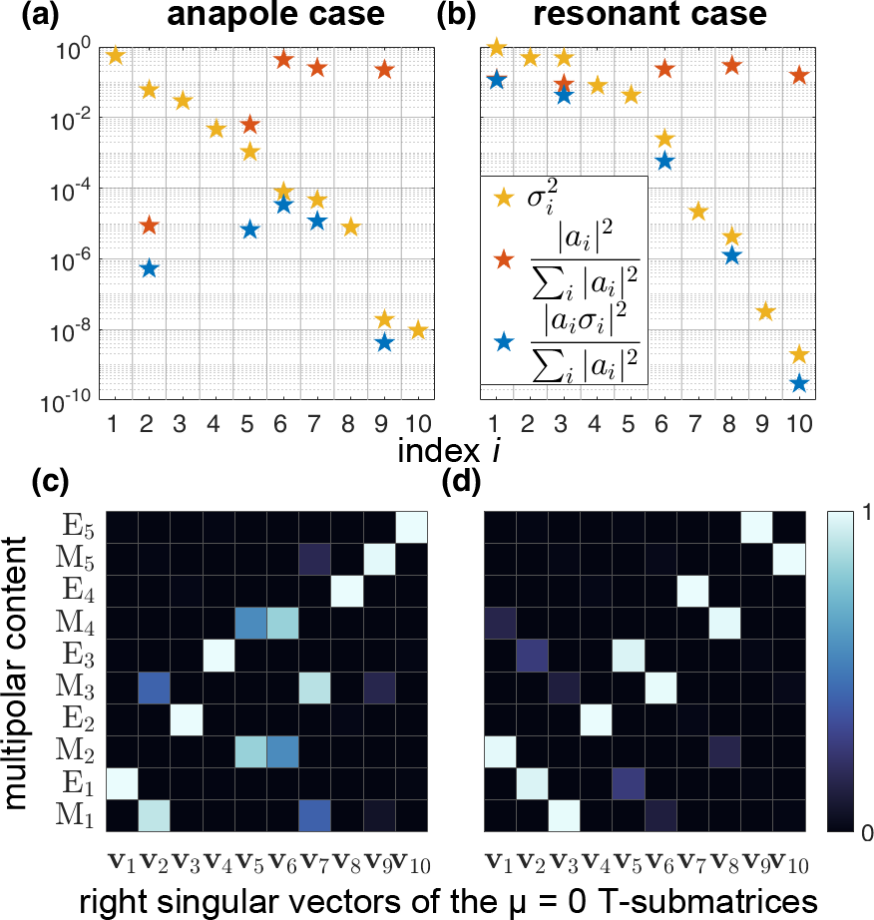
Comparison of the scattering behavior of two disks of the same aspect ratio but of different size under the prism of the singular value decomposition of their μ = 0 T-submatrices. One of them corresponds to an anapole case (a,c) and the other to a resonant case (b,d). In (a,b) we plot the singular values σ*_i_* of the T-submatrices (yellow stars), together with the coupling coefficients *a_i_* of the excitation field with the corresponding right singular vectors (red stars), as well as the final contribution of the correspondent singular mode to the total scattered power (blue stars). In (c,d) the multipolar content of each right singular vector **v***_i_* of the two T-submatrices is plotted. Weak coupling of the external field with the first right singular vectors of the T-submatrix, that correspond to high singular values, leads to the excitation of the anapole state.

In Figure 3a,b we plot the quantities 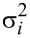 and 
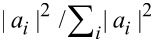
, as well as their product 
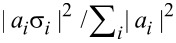
 for the two cylinders. As we mentioned above, the sum of the last quantity over the index *i* is proportional to the total scattered power. Therefore its minimization would lead to an anapole state. We can observe that for the cylinder that hosts an anapole state, there is weak coupling of the excitation field with the right-singular vectors that correspond to the first, largest, singular values of the matrix. The result is suppressed scattering leading to an anapole state. This does not happen in the case of the resonant cylinder. For the anapole case, the strongest part of the scattered power derives from the part of the incident field that corresponds to the sixth right singular vector, and is more than three orders of magnitude weaker than the prevailing scattered power that belongs to the first right singular vector of the T-submatrix of the disk in the resonant case.

Figure 3c,d depicts the multipolar content of the right singular vectors of the T-submatrices that belong to each of those two cylinders. As one can see, there is absolutely no coupling at all with the right singular vectors that have a multipolar content of electric type. In both cases there are no right singular vectors of mixed type multipolar content since for μ = 0 the T-submatrix of a rotationally symmetric particle is diagonal over the indicator α, which represents the type of the multipoles. Furthermore, due to the mirror symmetry of the particles, we can also observe that the right singular vectors are separated into vectors with either purely even or purely odd multipolar content.

Hence, we can claim that the key for the excitation of this particular nonradiating anapole state is connencted to the suppression of the coupling of the external field that we apply, with the first, third and fourth right singular vectors of the T-submatrix of the particle, which have electric multipolar content that is not supported by an azimuthally polarized focused beam. In addition, it is connected with the coincidence of the weak coupling of the excitation with the second right singular vector, which has a multipolar content of magnetic type and corresponds to a significantly large singular value that could potentially spoil the excitation of the anapole state.

## Conclusion

In this paper we describe the so-called magnetic anapole modes and discuss various experimental setups of how they can be obtained. Such modes are associated with the complete suppression of magnetic dipole scattering. In order to be able to obtain anapole states experimentally, we employed structured-light excitation of a particular configuration. Usually we use illumination with a plane wave at normal incidence. Such an excitation carries a multipolar content of μ = ±1 angular momentum and acts on the correspondent T-submatrices when it comes to scattering by rotationally symmetric particles. By using a rotationally symmetric excitation, instead, we have access to the μ = 0 T-submatrix of these particles, which constitutes a whole new scattering system, a whole new field to work with where new interesting phenomena may wait to be unveiled. It turns out that azimuthally polarized beams contain only spherical harmonics of magnetic type in the focal region. Depending on the size of the particle compared to the incident wavelength it can couple to dipole and/or higher-order harmonics. By using two counterpropagating out of phase beams it is possible to cancel out all harmonics of even order because of symmetry properties. Together with a phase mask applied to the beams, designated to suppress the interfering octupolar content, this provides an ideal condition for the excitation of a magnetic anapole state in a silicon nanosphere. We also discussed realistic setups, based on silicon nanodisks and nanopillars, which can be used for the experimental detection of magnetic anapole states. We also explained, under the T-matrix formalism, the physical mechanism of their excitation in the hosting nanoparticles, by means of a singular value decomposition of their T-matrices.

## Supporting Information

Supporting Information features the proof for the multipolar decomposition of an arbitrary plane wave and the formulas with which the elements of the T-matrix are calculated based on the EBCM method. It also includes a plot of the proposed phase mask for Figure 1c, electric and magnetic field plots corresponding to the anapole condition discussed in Figure 1b and Figure 1c, and some extra 2D plots of the multipolar decomposition of the scattered field of cylinders of various geometries illuminated by an azimuthally polarized, rotationally symmetric, focused beam.

File 1 Additional computational data.
