# Supplementary material for: Excitation of nonradiating magnetic anapole states with azimuthally polarized vector beams
Source: Beilstein J Nanotechnol. 2018 May 17;9:1478–90. doi: 10.3762/bjnano.9.139 (PMC6009353; doi:10.3762/bjnano.9.139)
Supplement: File 1 — Additional computational data. [file Beilstein_J_Nanotechnol-09-1478-s001.pdf]

Supporting Information

for

Excitation of nonradiating magnetic anapole states with azimuthally polarized vector beams

Aristeidis G. Lamprianidis*[1,2] and Andrey E. Miroshnichenko[3]

Address: [1]Nonlinear Physics Centre, The Australian National University, Canberra ACT 2601, Australia; [2]Department of Mathematics and Applied Mathematics, University of Crete, 70013 Heraklion, Crete, Greece and [3]School of Engineering and Information Technology, University of New South Wales, Canberra, ACT 2600, Australia

Email: Aristeidis G. Lamprianidis - aris.lamprianidis@anu.edu.au

* Corresponding author

Additional computational data

Multipolar decomposition of an arbitrary plane wave in vector spherical harmonics

The electric field produced by an electric dipole $\mathbf{p}$ located at $\mathbf{r_0}$ is given by the formula below:

$$\mathbf{E}(\mathbf{r}) \;=\; \frac{\mathrm{k}_0^2}{\varepsilon_0}\overset{\leftrightarrow}{\mathbf{G}}(\mathbf{r},\mathbf{r_0})\mathbf{p}(\mathbf{r_0}). \tag{S1}$$

Avoiding the singularity at $\mathbf{r} = \mathbf{r_0}$ the dyadic Green function above can be decomposed into a series of dyadic products of vector spherical harmonics in the following way[1]:

$$\overset{\leftrightarrow}{\mathbf{G}}(\mathbf{r},\mathbf{r_0}) \;=\; \begin{cases} \mathrm{jk}_0 \sum\limits_{\nu\mu,\alpha} (-1)^{\mu}\mathbf{F}^{(1)}_{\alpha,\mu\nu}(\mathrm{k}_0\mathbf{r})\mathbf{F}^{(3)}_{\alpha,-\mu\nu}(\mathrm{k}_0\mathbf{r_0}), & \text{for } \mathrm{r} < \mathrm{r}_0 \\ \mathrm{jk}_0 \sum\limits_{\nu\mu,\alpha} (-1)^{\mu}\mathbf{F}^{(3)}_{\alpha,\mu\nu}(\mathrm{k}_0\mathbf{r})\mathbf{F}^{(1)}_{\alpha,-\mu\nu}(\mathrm{k}_0\mathbf{r_0}), & \text{for } \mathrm{r} > \mathrm{r}_0 \end{cases} \tag{S2}$$

We consider a dipole at infinity: $\mathrm{k}_0\mathrm{r}_0 \to \infty$ and by applying the large argument approximation of the spherical Hankel functions: $z^{(3)}_{\alpha,\nu}(x) \xrightarrow{x>>1} \frac{e^{\mathrm{j}x}}{x}(-\mathrm{j})^{\nu+\delta_{\alpha\mathrm{M}}}$ and rejecting the $\mathrm{O}(\frac{1}{\mathrm{r}_0^2})$ radial term as negligible, we end up with the following far field approximation of the dyadic Green function that describes the field that a dipole at infinity, and in a direction given by the unit vector $\hat{\mathrm{r}}_0(\theta_0,\phi_0) = \frac{\mathbf{r_0}}{\mathrm{r}_0}$, produces around the center of the coordinate system:

$$\overset{\leftrightarrow}{\mathbf{G}}_{ff}(\mathbf{r},\mathbf{r_0}) \;=\; \mathrm{jk}_0 \sum_{\nu\mu,\alpha} (-1)^{\mu}\mathbf{F}^{(1)}_{\alpha,\mu\nu}(\mathrm{k}_0\mathbf{r})\left[\mathbf{F}^{(3)}_{\alpha,-\mu\nu}(\mathrm{k}_0\mathbf{r_0})\right]^{ff}, \tag{S3}$$

where

$$\begin{aligned}\left[\mathbf{F}^{(3)}_{\alpha,-\mu\nu}(\mathrm{k}_0\mathbf{r_0})\right]^{ff} \;&=\; \frac{e^{\mathrm{jk}_0\mathrm{r}_0}}{\mathrm{k}_0\mathrm{r}_0}(-\mathrm{j})^{\nu}\mathbf{f}_{\alpha,-\mu\nu}(\hat{\mathrm{r}}_0) \\ &=\; \frac{e^{\mathrm{jk}_0\mathrm{r}_0}}{\mathrm{k}_0\mathrm{r}_0}(-\mathrm{j})^{\nu}\gamma_{-\mu\nu}\left[\hat{\theta}_0\tau^{(2-\delta_{\alpha\mathrm{M}})}_{-\mu\nu}(\theta_0) + \mathrm{j}\hat{\phi}_0\tau^{(2-\delta_{\alpha\mathrm{N}})}_{-\mu\nu}(\theta_0)\right]e^{-\mathrm{j}\mu\phi_0},\end{aligned} \tag{S4}$$

On the other hand, the far-field approximation of the dyadic Green function can also be written as [2]:

$$\overleftrightarrow{\mathbf{G}}_{ff}(\mathbf{r},\mathbf{r_0}) = \frac{e^{\mathrm{jk_0r'}}}{4\pi\mathrm{r'}}\left(\overleftrightarrow{\mathbf{I}} - \hat{\mathrm{r}}'\hat{\mathrm{r}}'\right), \tag{S5}$$

where $\mathbf{r}' = \mathbf{r} - \mathbf{r_0}$. If we also restrict again our observation around the center of the coordinate system, we can apply the approximation $\mathrm{r}' \simeq \mathrm{r_0} + \mathbf{r}\cdot\hat{\mathrm{r}}'$ for the exponential, whereas the approximation $\mathrm{r}' \simeq \mathrm{r_0}$ is sufficient for the denominator. Moreover, $\hat{\mathrm{r}}' \simeq -\hat{\mathrm{r}}_0$. So, the previous expression, under the approximations above, evolves into the following formula:

$$\overleftrightarrow{\mathbf{G}}_{ff}(\mathbf{r},\mathbf{r_0}) = \frac{e^{\mathrm{jk_0r_0}}}{4\pi\mathrm{r_0}}\left(\hat{\theta}_0\hat{\theta}_0 + \hat{\phi}_0\hat{\phi}_0\right)e^{-\mathrm{jk_0}\hat{\mathrm{r}}_0\cdot\mathbf{r}}. \tag{S6}$$

First we define $(\gamma,\delta) = (\pi+\phi_0, \pi-\theta_0)$ and therefore we have

$$\left(\hat{\mathrm{r}}_0, \hat{\theta}_0, \hat{\phi}_0\right) = \left(-\hat{\mathrm{r}}(\gamma,\delta), \hat{\theta}(\gamma,\delta), -\hat{\phi}(\gamma,\delta)\right) = \left(-\hat{\mathrm{i}}(\gamma,\delta), \hat{e}_{\hat{\rho}}(\gamma,\delta), -\hat{e}_{\hat{\phi}}(\gamma,\delta)\right).$$

Then, by comparison of Equation 3 and Equation 6, we can finally take the spherical amplitudes $\dot{\mathcal{A}}_{\alpha,\mu\nu}(\hat{\mathrm{i}},\hat{\mathrm{e}}_\mathrm{p})$ of the multipolar decomposition of an arbitrary plane wave of direction of propagation $\hat{\mathrm{i}}(\gamma,\delta)$ and polarization $\hat{\mathrm{e}}_\mathrm{p}(\gamma,\delta)$ in vector spherical harmonics:

$$\hat{\mathrm{e}}_\mathrm{p}(\gamma,\delta)\,e^{\mathrm{jk_0}\hat{\mathrm{i}}(\gamma,\delta)\cdot\mathbf{r}} = \sum_{\nu\mu,\alpha}\dot{\mathcal{A}}_{\alpha,\mu\nu}(\hat{\mathrm{i}},\hat{\mathrm{e}}_\mathrm{p})\mathbf{F}^{(1)}_{\alpha,\mu\nu}(\mathrm{k_0}\mathbf{r}), \tag{S7}$$

where the indicator p acquires the names $\hat{\rho},\hat{\phi}$ to refer to $\hat{\theta}$(TM)- and $\hat{\phi}$(TE)-polarized waves, respectively, and

$$\dot{\mathcal{A}}_{\alpha,\mu\nu}(\hat{\mathrm{i}},\hat{\mathrm{e}}_{\mathrm{p}}) \;=\; 4\pi \mathrm{j}^{-\nu+\delta_{\mathrm{p}\hat{\rho}}}\,\gamma_{-\mu\nu}\,\tau^{(2-\Delta)}_{-\mu\nu}(\pi-\delta)\,e^{-\mathrm{j}\mu\gamma}, \tag{S8}$$

where $\Delta = \delta_{\alpha\mathrm{N}}\delta_{\mathrm{p}\hat{\phi}} + \delta_{\alpha\mathrm{M}}\delta_{\mathrm{p}\hat{\rho}}$ and $\delta_{\alpha\beta}$ is the Kronecker delta that yields 1, for $\alpha = \beta$, or 0, for $\alpha \neq \beta$. This last formula actually constitutes the expansion of the cartesian eigensolutions of the Helmholtz equation of free space, that is the plane waves, over the basis of the spherical eigensolutions of it, that is the vector spherical harmonics.

EBCM method for calculation of the elements of the T-matrix of a rotationally symmetric, star-shaped, homogeneous particle

We consider a particle with optical properties $\varepsilon_1, \mu_1, \mathrm{k}_1$ inside an infinite medium with optical properties $\varepsilon_0, \mu_0, \mathrm{k}_0$. The surface of the particle S_1 is given as a function of the polar and the azimuthal angles $\mathrm{r}_1'(\theta_1, \phi_1)$ in the natural frame of the scatterer. We also define the quantities $x_0' = \mathrm{k}_0\mathrm{r}_1'$, $x_1' = \mathrm{k}_1\mathrm{r}_1'$. According to the EBCM method [1] the spherical amplitudes of the incident, $\mathcal{A}_{\alpha,\mu\nu}$, and the scattered, $\mathcal{B}_{\alpha,\mu\nu}$, field are connected with the spherical amplitudes, $\mathcal{C}_{\alpha,\mu\nu}$, of the field induced inside the particle, $\mathbf{E}_1(\mathbf{r}_1) = \sum\limits_{\nu\mu,\alpha} \mathcal{C}_{\alpha,\mu\nu}\mathbf{F}^{(1)}_{\alpha,\mu\nu}(\mathrm{k}_1\mathbf{r}_1)$, via the $\mathbf{Q}^3$ and $\mathbf{Q}^1$ matrices respectively:

$$\begin{aligned}
\mathcal{A}_{\alpha,\mu\nu} &= \sum_{\nu'\mu',\alpha'} \mathbf{Q}^{\alpha,\mu\nu;(3)}_{\alpha',\mu'\nu'}\mathcal{C}_{\alpha',\mu'\nu'}, && \text{(S9)}\\
\mathcal{B}_{\alpha,\mu\nu} &= \sum_{\nu'\mu',\alpha'} \mathbf{Q}^{\alpha,\mu\nu;(1)}_{\alpha',\mu'\nu'}\mathcal{C}_{\alpha',\mu'\nu'}, && \text{(S10)}\\
& && \text{(S11)}
\end{aligned}$$

or in a matrix formulation: $\mathbf{A} = \mathbf{Q}_3 \cdot \mathbf{C}$, $\mathbf{B} = \mathbf{Q}_1 \cdot \mathbf{C}$. Therefore, the T-matrix will be given by the following formula

$$\mathbf{T} = \mathbf{Q}_1\mathbf{Q}_3^{-1}. \tag{S12}$$

The elements of the $\mathbf{Q}_\mathrm{q}$ matrices, with index q taking the values of 1 or 3, can be calculated by the following integrals over the surface of the particle:

$$\mathbf{Q}^{\alpha,\mu\nu;(\mathrm{q})}_{\alpha',\mu'\nu'} = \mathrm{j}^{\mathrm{q}+2\mu}\mathrm{k}_0^2 \oint_{S_1} \mathbf{n}\cdot\left\{\frac{\mu_0\mathrm{k}_1}{\mu_1\mathrm{k}_0}\mathbf{F}^{(1)}_{\beta',\mu'\nu'}\left(\mathrm{k}_1\mathbf{r}_1'\right)\times\mathbf{F}^{(\mathrm{q})}_{\alpha,-\mu\nu}(\mathrm{k}_0\mathbf{r}_1') + \mathbf{F}^{(1)}_{\alpha',\mu'\nu'}\left(\mathrm{k}_1\mathbf{r}_1'\right)\times\mathbf{F}^{(\mathrm{q})}_{\beta,-\mu\nu}(\mathrm{k}_0\mathbf{r}_1')\right\}\mathrm{d}s', \tag{S13}$$

where $\alpha \neq \beta$, $\alpha' \neq \beta'$, $\mathbf{n}$ is the perpendicular to the surface of the particle unitary vector and $\mathrm{d}s'\mathbf{n}(\mathbf{r}'_1) = \left[\mathrm{r}'^2_1 \sin\theta_1 \hat{\mathrm{r}}_1 - \mathrm{r}'_1 \sin\theta_1 \frac{\partial \mathrm{r}'_1}{\partial \theta_1} \hat{\theta}_1 - \mathrm{r}'_1 \frac{\partial \mathrm{r}'_1}{\partial \phi_1} \hat{\phi}_1\right] \mathrm{d}\theta_1 \mathrm{d}\phi_1$.

For a rotationally symmetric particle $\left(\frac{\partial \mathrm{r}'_1}{\partial \phi_1} = 0\right)$ we can perform the integration over the azimuthal angle analytically and therefore we end up with the simplified formulas

$$\left[\mathrm{Q}^{\mathrm{M},\mu\nu;(\mathrm{q})}_{\mathrm{M},\mu'\nu'}\right]^{\mathrm{rs}} = \mathrm{j}\frac{\mu_0 k_1}{\mu_1 k_0}\mathrm{Q1}^{\mu\nu;(\mathrm{q})}_{\mu'\nu'} - \mathrm{j}\mathrm{Q2}^{\mu\nu;(\mathrm{q})}_{\mu'\nu'} \tag{S14}$$

$$\left[\mathrm{Q}^{\mathrm{N},\mu\nu;(\mathrm{q})}_{\mathrm{N},\mu'\nu'}\right]^{\mathrm{rs}} = -\mathrm{j}\frac{\mu_0 k_1}{\mu_1 k_0}\mathrm{Q2}^{\mu\nu;(\mathrm{q})}_{\mu'\nu'} + \mathrm{j}\mathrm{Q1}^{\mu\nu;(\mathrm{q})}_{\mu'\nu'} \tag{S15}$$

$$\left[\mathrm{Q}^{\mathrm{M},\mu\nu;(\mathrm{q})}_{\mathrm{N},\mu'\nu'}\right]^{\mathrm{rs}} = \frac{\mu_0 k_1}{\mu_1 k_0}\mathrm{Q3}^{\mu\nu;(\mathrm{q})}_{\mu'\nu'} + \mathrm{Q4}^{\mu\nu;(\mathrm{q})}_{\mu'\nu'} \tag{S16}$$

$$\left[\mathrm{Q}^{\mathrm{N},\mu\nu;(\mathrm{q})}_{\mathrm{M},\mu'\nu'}\right]^{\mathrm{rs}} = \frac{\mu_0 k_1}{\mu_1 k_0}\mathrm{Q4}^{\mu\nu;(\mathrm{q})}_{\mu'\nu'} + \mathrm{Q3}^{\mu\nu;(\mathrm{q})}_{\mu'\nu'} \tag{S17}$$

where

$$
\begin{aligned}
\mathrm{Q1}^{\mu\nu;(\mathrm{q})}_{\mu'\nu'} &= 2\pi\delta_{\mu\mu'}\Gamma^{\mu\nu;(\mathrm{q})}_{\mu'\nu'}\int_0^{\pi}\sin\theta_1 \mathrm{d}\theta_1 x_0' z^{(\mathrm{q})}_{\mathrm{M},\nu}(x_0')\times \\
&\times \left\{x_0'\left[\tau^{(2)}_{\mu'\nu'}(\theta_1)\tau^{(2)}_{-\mu\nu}(\theta_1)-\tau^{(1)}_{\mu'\nu'}(\theta_1)\tau^{(1)}_{-\mu\nu}(\theta_1)\right]z^{(1)}_{\mathrm{N},\nu'}(x_1')+\right. \\
&+ \left.\frac{\partial x_0'}{\partial\theta_1}\frac{\nu'(\nu'+1)}{x_1'}z^{(1)}_{\mathrm{M},\nu'}(x_1')\mathrm{P}^{\mu'}_{\nu'}(\cos\theta_1)\tau^{(2)}_{-\mu\nu}(\theta_1)\right\} \qquad \text{(S18)}
\end{aligned}
$$

$$
\begin{aligned}
\mathrm{Q2}^{\mu\nu;(\mathrm{q})}_{\mu'\nu'} &= 2\pi\delta_{\mu\mu'}\Gamma^{\mu\nu;(\mathrm{q})}_{\mu'\nu'}\int_0^{\pi}\sin\theta_1 \mathrm{d}\theta_1 x_0' z^{(1)}_{\mathrm{M},\nu'}(x_1')\times \\
&\times \left\{x_0'\left[\tau^{(2)}_{\mu'\nu'}(\theta_1)\tau^{(2)}_{-\mu\nu}(\theta_1)-\tau^{(1)}_{\mu'\nu'}(\theta_1)\tau^{(1)}_{-\mu\nu}(\theta_1)\right]z^{(\mathrm{q})}_{\mathrm{N},\nu}(x_0')+\right. \\
&+ \left.\frac{\partial x_0'}{\partial\theta_1}\frac{\nu(\nu+1)}{x_0'}z^{(\mathrm{q})}_{\mathrm{M},\nu}(x_0')\mathrm{P}^{-\mu}_{\nu}(\cos\theta_1)\tau^{(2)}_{\mu'\nu'}(\theta_1)\right\} \qquad \text{(S19)}
\end{aligned}
$$

$$
\mathrm{Q3}^{\mu\nu;(\mathrm{q})}_{\mu'\nu'} = 2\pi\delta_{\mu\mu'}\Gamma^{\mu\nu;(\mathrm{q})}_{\mu'\nu'}\int_0^{\pi}\sin\theta_1 \mathrm{d}\theta_1 x_0'^2 z^{(1)}_{\mathrm{M},\nu'}(x_1')z^{(\mathrm{q})}_{\mathrm{M},\nu}(x_0')\left[\tau^{(2)}_{\mu'\nu'}(\theta_1)\tau^{(1)}_{-\mu\nu}(\theta_1)-\tau^{(1)}_{\mu'\nu'}(\theta_1)\tau^{(2)}_{-\mu\nu}(\theta_1)\right] \qquad \text{(S20)}
$$

$$
\begin{aligned}
\mathrm{Q4}^{\mu\nu;(\mathrm{q})}_{\mu'\nu'} &= 2\pi\delta_{\mu\mu'}\Gamma^{\mu\nu;(\mathrm{q})}_{\mu'\nu'}\int_0^{\pi}\sin\theta_1 \mathrm{d}\theta_1 x_0'\left\{x_0' z^{(1)}_{\mathrm{N},\nu'}(x_1')z^{(\mathrm{q})}_{\mathrm{N},\nu}(x_0')\left[\tau^{(2)}_{\mu'\nu'}(\theta_1)\tau^{(1)}_{-\mu\nu}(\theta_1)-\tau^{(1)}_{\mu'\nu'}(\theta_1)\tau^{(2)}_{-\mu\nu}(\theta_1)\right]+\right. \\
&+ \left.\frac{\partial x_0'}{\partial\theta_1}\mathrm{P}^{\mu'}_{\nu'}(\cos\theta_1)\tau^{(1)}_{-\mu\nu}(\theta_1)\left[\frac{\nu(\nu+1)}{x_0'}z^{(1)}_{\mathrm{N},\nu'}(x_1')z^{(\mathrm{q})}_{\mathrm{M},\nu}(x_0')+\frac{\nu'(\nu'+1)}{x_1'}z^{(1)}_{\mathrm{M},\nu'}(x_1')z^{(\mathrm{q})}_{\mathrm{N},\nu}(x_0')\right]\right\}, \qquad \text{(S21)}
\end{aligned}
$$

where $\Gamma^{\mu\nu;(\mathrm{q})}_{\mu'\nu'} = \mathrm{j}^{\mathrm{q}+1}(-1)^{\mu}\gamma_{-\mu\nu}\gamma_{\mu'\nu'}$.

Finally, for the particular case of a rotationally symmetric cylindrical disk of height h, diameter d and aspect ratio $\mathrm{A_r} = \frac{\mathrm{d}}{\mathrm{h}}$, the surface of the particle will be given by the function

$$
\mathrm{r}_1'(\theta_1) = \begin{cases} \frac{\mathrm{h}}{2\cos\theta_1}, & \text{for} \quad \theta_1 \in [0, \tan^{-1}(\mathrm{A_r})] \\ \frac{\mathrm{d}}{2\sin\theta_1}, & \text{for} \quad \theta_1 \in [\tan^{-1}(\mathrm{A_r}), \pi - \tan^{-1}(\mathrm{A_r})] \\ \frac{-\mathrm{h}}{2\cos\theta_1}, & \text{for} \quad \theta_1 \in [\pi - \tan^{-1}(\mathrm{A_r}), \pi] \end{cases} \qquad \text{(S22)}
$$

with its partial derivative over the polar angle being

$$\frac{\partial \mathrm{r}_1'(\theta_1)}{\partial \theta_1} = \begin{cases} \frac{\mathrm{h}\sin\theta_1}{\cos 2\theta_1 + 1}, & \text{for} \quad \theta_1 \in [0, \tan^{-1}(\mathrm{A_r})] \\ \frac{\mathrm{d}\cos\theta_1}{\cos 2\theta_1 - 1}, & \text{for} \quad \theta_1 \in [\tan^{-1}(\mathrm{A_r}), \pi - \tan^{-1}(\mathrm{A_r})] \\ \frac{-\mathrm{h}\sin\theta_1}{\cos 2\theta_1 + 1}, & \text{for} \quad \theta_1 \in [\pi - \tan^{-1}(\mathrm{A_r}), \pi] \end{cases} \tag{S23}$$

Additional plots supporting the results of Figure 1 of the main manuscript

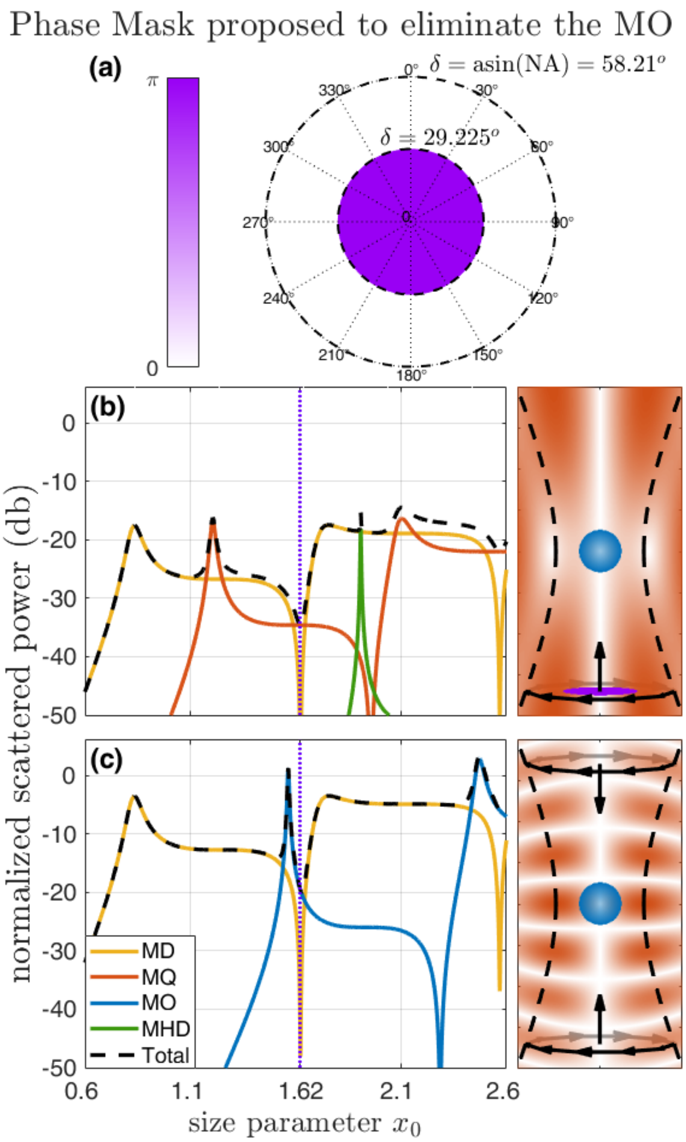

Figure S1: a) Phase mask proposed to eliminate the magnetic octupole interference that hinders the access to the anapole condition. b,c) Multipolar decomposition of the normalized scattered power $P_{\text{sca}}/P_{\text{inc}}$ corresponding to two illumination schemes: single beam excitation with phase mask applied and excitation with two out-of-phase beam, respectively, the combination of which gives us the excitation of the ideal magnetic anapole of Figure 1c of the paper.

Field plots that correspond to Figure 1b,c of the main manuscript

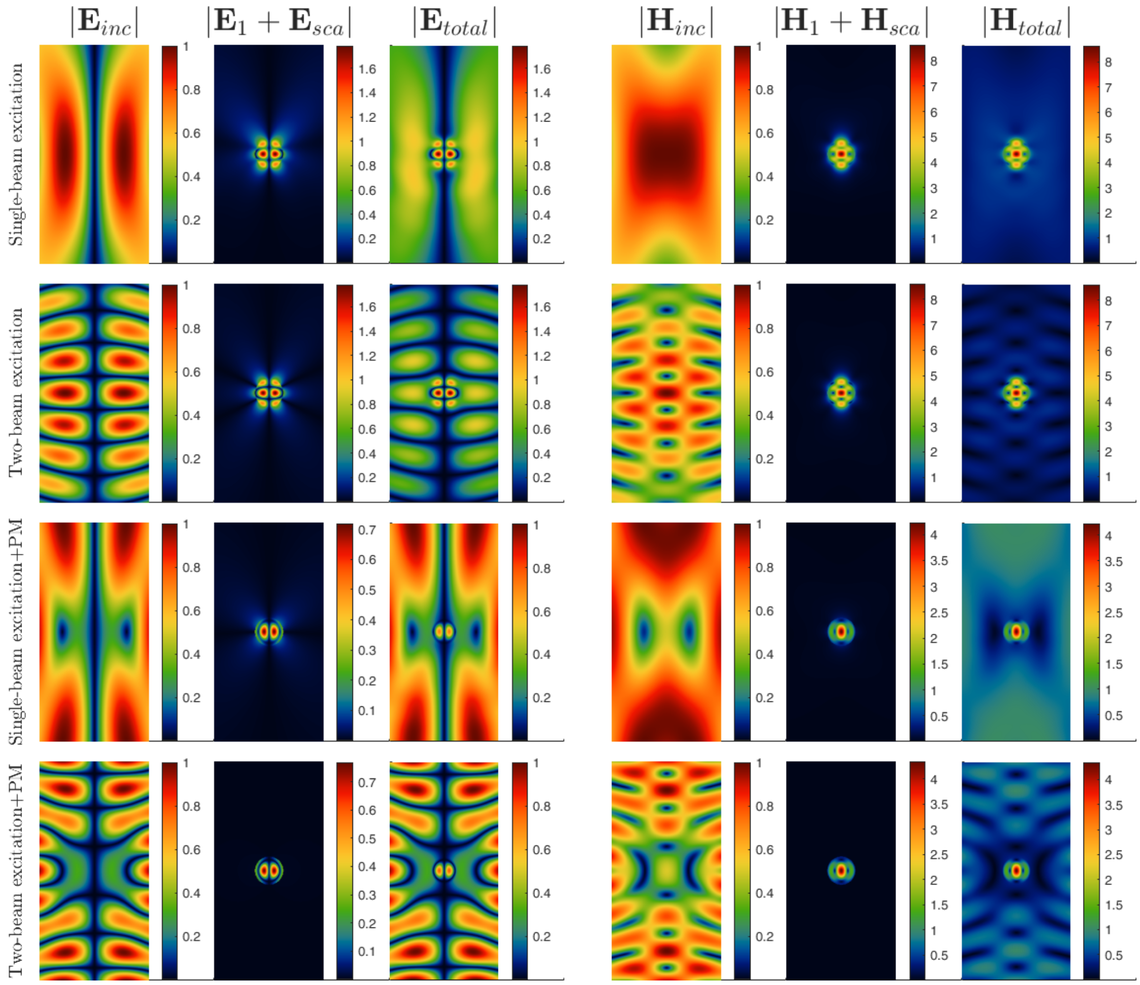

Figure S2: Normalized Electric and Magnetic field intensity plots that refer to single-and two-beam excitation schemes, with and without the proposed phase modulation, of a silicon sphere of size parameter $x_0 = 1.62$ placed at the focal point and corresponding to the anapole condition case. The field plots are over a region of 2×4 wavelengths at the focal area on the ρOz plane. Under the single beam excitation scheme we have both quadrupole and octupole terms spoiling the anapole condition. Adding a second, out of phase, counterpropagating beam we manage to kill the quadrupole interference, and, moreover, applying the proposed phase mask we elliminate also the octupole interference, leading finally to an ideal magnetic anapole excitation. The major price that we pay is that the phase mask weakens the field intensity at the vicinity of the focal point.

Multipolar decomposition of the normalized scattered power that corresponds to Figure 2a of the main manuscript

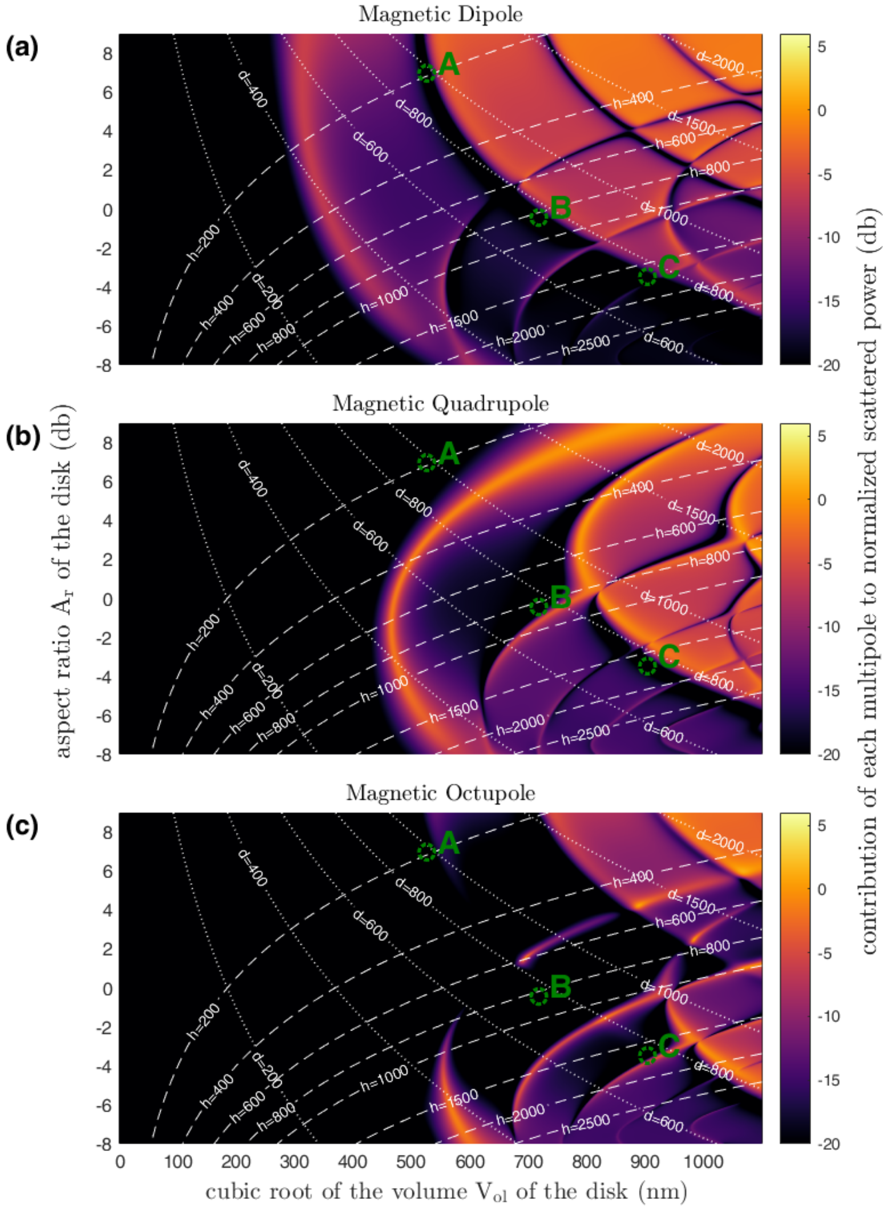

Figure S3: Multipolar decomposition of the normalized scattered power P_{sca}/P_{inc} that corresponds to Figure 2a of the paper. a) Magnetic dipole contribution. b) Magnetic quadrupole contribution. c) Magnetic octupole contribution.

References

1. Mishchenko, M.; D. Travis, L.; Lacis, A. *Scattering, Absorption, and Emission of Light by Small Particles*; 2002; Vol. 4.

2. Novotny, L.; Hecht, B. *Principles of Nano-Optics*, 2nd ed.; Cambridge University Press, 2012; doi:10.1017/CBO9780511794193.
